# Supplementary material for: The RGD-binding integrins αvβ6 and αvβ8 are receptors for mouse adenovirus-1 and -3 infection
Source: PLoS Pathog. 2021 Dec 15;17(12):e1010083. doi: 10.1371/journal.ppat.1010083 (PMC8673666; doi:10.1371/journal.ppat.1010083)
Supplement: S1 Table — (DOCX) [file ppat.1010083.s021.docx]

S1 Table. Determination molar mass and mass fraction of knob preparations by SEC-MALS.

|  | FKb-M1 | FK-M2 | FK-M3 | FKb-H5 |
| --- | --- | --- | --- | --- |
| theoretical molar mass (kDa)  monomer | 31.2 | 33.3 | 30.9 | 24.3 |
| determined molar mass (kDa)  main peak  mass fraction (%) | 93.3  78.9 | 86.2  98 | 90.8  89.3 | 71.3  100 |
| secondary peak  mass fraction (%) | 31.8  18.3 |  | 188.4  5.8 |  |
