# Supplementary material for: The RGD-binding integrins αvβ6 and αvβ8 are receptors for mouse adenovirus-1 and -3 infection
Source: PLoS Pathog. 2021 Dec 15;17(12):e1010083. doi: 10.1371/journal.ppat.1010083 (PMC8673666; doi:10.1371/journal.ppat.1010083)
Supplement: S2 Table — (DOCX) [file ppat.1010083.s022.docx]

S2 Table. Oligonucleotides used for generation of BACmid and and knock out constructs.

| virus BACmid  construct/ gRNA | Description | Oligo  Nr | Oligo-Sequence (5’-3’) |
| --- | --- | --- | --- |
| pKSB2-M1 | left terminal M1 | 939 | GATCGCGGCCGCGTTTAAACATCATCAATAAT ATACAGTTAGC |
|  |  | 940 | TTCTAGAGCTAGCCATTGCGAGTAGCTTCCTGAGCTGC |
|  | right terminal M1 | 941 | TTCTAGAACTAGTACAGCTCCGCCACACAGCGAACGG |
|  |  | 942 | GGGATCCGTTTAAACATCATCAA TAATATACAGTTAGC |
|  | Zeo cassette | 874 | GACTAGTTTTTCGGATCTGATCAGCACG |
|  |  | 875 | GACTAGTGGAAAACGATT CCGAAGCCC |
| pKSB2-M1-∆E1A-GFP | galK cassette | 947 | TTTGTTTGTTATTTGGTGCGATTTTTCGACTTTTGACTCACCTGTTGACAATTAATCATCGGCA |
|  |  | 952 | GTACTAACATAACAGCTGCCAAAAAACAACAATACAACCTCTCAGCACTGTCCTGCTCCTT |
|  | GFP ORF | 950 | TTTGTTTGTTATTTGGTGCGATTTTTCGACTTTTGACTCATCGCCACCATGGTGAGCAAG |
|  |  | 951 | GTACTAACATAACAGCTGCCAAAAAACAACAATACAACCTTTCTTTCCCACCCTTAAGCC |
| pKSB2-M1-IX-G | galK cassette | 2325 | GAGGACGGAGCTGAAGACATTGAGGAAAACGGGGAAGAAAGTGATCCTGTTGACAATTAATCATCGGCA |
|  |  | 2326 | TTTTTATTAAACATAAAGCGCGTGAGCATGCATCTTTATTTGGGACTCAGCACTGTCCTGCTCCTT |
|  | 2A-GFP ORF | 2327 | GAGGACGGAGCTGAAGACATTGAGGAAAACGGGGAAGAAAGTGATAGAAAAAGAAGGGCACCGGTG |
|  |  | 2553 | TTTTTATTAAACATAAAGCGCGTGAGCATGCATCTTTATTTGGGATTACTTGTACAGCTCGTCCATGC |
| pBl-2A-GFP | 2A-GFP ORF | 2305 | CGGggtaccAGAAAAAGAAGGgcaccggtgaaacagactttgaattttgaccttctcaagttggcaggagacgttgagtccaaccctgggcccATGGTGAGCAAGGGCGAGGAG |
|  |  | 2306 | ATGCATGCgcggccgcTTACTTGTACAGCTCGTCCATG |
| pKSB2-SceI-KanR | KanR | 2371 | CAAAATACCTGGTTACCTAGGGATAACAGGGTAATATGCATCCTTAATTAAGGGCGGCCGCTGAGGTCTGCCTCGTGAAGAAG |
|  |  | 2372 | GCGCATTTAAATATTACCCTGTTATCCCTAAAGCTTGGTACCGCGGATCCTGAGGTCTGCCTCGTGAAGAAG |
| pKSB2-M2 | left terminal M2 | 1320 | GATCGCGGCCGCATATGTTAATTAACTTCTTCATCTAATTAAAAATAGAA |
|  |  | 1321 | AATTCTAGAGGGTAAAAGCACAGACATGGT |
|  | right terminal M2 | 1322 | TATTCTAGACTAGTCGCGCTGCCTGCCTATATACCC |
|  |  | 1323 | ATGGGATCCATATGTTAATTAACTTCTTCATCTAATTAAAAATAG |
| pKSB2-M2-∆E1A-G | galK cassette | 1597 | AGAGTGGCCAACTCTTGAGTGTGTGCGAGAAGAGTTTTCTCCGCGCCTGTTGACAATTAATCATCGGCA |
|  |  | 1598 | AGTACACACTAACCCACATATCCGGGGTCGTGGCGTTCGACTCACCTCAGCACTGTCCTGCTCCTT |
|  | 2A-GFP ORF | 1717 | AGAGTGGCCAACTCTTGAGTGTGTGCGAGAAGAGTTTTCTCCGCGTCGCCACCATGGTGAGCAAG |
|  |  | 1718 | AGTACACACTAACCCACATATCCGGGGTCGTGGCGTTCGACTCACCTTCTTTCCCACCCTTAAGCC |
| pKSB2-SceI-M3-∆E1A-G | left terminal M3 | 2090 | AAGCTgagctcATGCATttaaatTAATTAAcatcatcaataatatacacatg |
|  |  | 2091 | gcTCTAGAggaaaaacaatgttaaccctc |
|  | right terminal M3 | 2092 | gcTCTAGActagttttaggactttacgagcgatc |
|  |  | 2093 | cgcgAAGCTTatttaaaTTAATTAAcatcatcaataatatacacatg |
|  | galK cassette | 2125 | AACCTCGTACCCGCTTTTTCTGTTTTTTTACTCTTTGCTTTGACACCTGTTGACAATTAATCATCGGCA |
|  |  | 2126 | CTATGAATAAAGAATAGCAAGCAAGAAGCCAAAAAGGAAACTTACCTCAGCACTGTCCTGCTCCTT |
|  | GFP ORF | 2127 | AACCTCGTACCCGCTTTTTCTGTTTTTTTACTCTTTGCTTTGACAGCCACCATGGTGAGCAAG |
|  |  | 2128 | CTATGAATAAAGAATAGCAAGCAAGAAGCCAAAAAGGAAACTTACTTCTTTCCCACCCTTAAGCC |
| pKSB2-SceI-M3-IX-G | galK cassette | 2556 | CAGAGACTAAGTGGAGAGGGAGAGTCGGAAGGGGAGGAGAGTGACCCTGTTGACAATTAATCATCGGCA |
|  |  | 2557 | ATGTGGGTCAGTTATTTCAAAAGCATGTGAATTAAAGAATATGGGCTCAGCACTGTCCTGCTCCTT |
|  | FS2A-GFP ORF | 2560 | CAGAGACTAAGTGGAGAGGGAGAGTCGGAAGGGGAGGAGAGTGACAGAAAAAGAAGGGCACCGGTGAAAC |
|  |  | 2561 | ATGTGGGTCAGTTATTTCAAAAGCATGTGAATTAAAGAATATGGGTTACTTGTACAGCTCGTCCATGC |
| pKSB2-H5 | left terminal M2 | 943 | ATAAGAATGCGGCCGCATTTAAATTAATTAACATCATCAATAATATACCTTATTTTGGATTG |
|  |  | 958 | GCTCTAGAGCTAGACTATAATAATAAAACGCCAACTTTG |
|  | right terminal | 945 | GCTCTAGAACTAGTCAGCCTTACCAGTAAAAAAGAAAACC |
|  |  | 946 | CCCAAGCTTAATTAATTTAAATCATCATCAATAATATACCTTATTTTGGATTG |
| pKSB2-H5-∆E3B-CG | galK cassette | 2910 | ACTGAAACACATGTTCTTTTCTCTTACAGTATGATTAAATGAGACCCTGTTGACAATTAATCATCGGCA |
|  |  | 2911 | GACAGAAATTTGCTAACTGATTTTAAGTAAGTGATGCTTTATTATCTCAGCACTGTCCTGCTCCTT |
|  | CMV-GFP cassette | 2912 | ACTGAAACACATGTTCTTTTCTCTTACAGTATGATTAAATGAGACTAGTTATTAATAGTAATCAATTACGGGGTC |
|  |  | 2913 | GACAGAAATTTGCTAACTGATTTTAAGTAAGTGATGCTTTATTATTTAAGATACATTGATGAGTTTGGACAAACC |
| pKSB2-H5-∆E3B-CG-FK-M1 | galK cassette | 2903 | AGCACAGGTGCCATTACAGTAGGAAACAAAAATAATGATAAGCTACCTGTTGACAATTAATCATCGGCA |
|  |  | 2900 | TGCAATTGAAAAATAAACACGTTGAAACATAACACAAACGATTCTCTCAGCACTGTCCTGCTCCTT |
|  | fiber knob M1 | 2904 | AGCACAGGTGCCATTACAGTAGGAAACAAAAATAATGATAAGCTAACACTGTGGACAGGCCTTCC |
|  |  | 2902 | TGCAATTGAAAAATAAACACGTTGAAACATAACACAAACGATTCTTTAATAGTCTTCAGCATAGTACCAAAAAC |
| pKSB2-H5-∆E3B-CG-FK-M3 | fiber knob M3 | 2917 | AGCACAGGTGCCATTACAGTAGGAAACAAAAATAATGATAAGCTAACCATATGGACAGGTATACCCATAGC |
|  |  | 2918 | TGCAATTGAAAAATAAACACGTTGAAACATAACACAAACGATTCTTTAATAGTCTTCTGCCAGATACCAGAAAC |
| pKSB2-H35 | left  terminal | 1040 | ATAAGAATGCGGCCGCATTTAAATGTTTAAACATCATCAATAATATACCTTATAG |
|  |  | 1041 | GCTCTAGACAGTCTCAGCAGAGATTATTTC |
|  | right terminal | 1042 | GCTCTAGAACTAGTGACCAGCATGAATAAGTC |
|  |  | 1043 | CTCCCAAGCTTATTTAAATGTTTAAACCATCATCAATAATATACCTTATAG |
| pKSB2-H35-∆E1-CG | galK  cassette | 1052 | GTACCGTGTCAAAGTCTTCTGTTTTTACGTAGGTGTCAGCCCTGTTGACAATTAATCATCGGCA |
|  |  | 1053 | CGCATGCGCGCACCCTCGATCTCGTATCATCATACCTCAGCTCAGCACTGTCCTGCTCCTT |
|  | CMV-GFP cassette | 1054 | GTACCGTGTCAAAGTCTTCTGTTTTTACGTAGGTGTCAGGCCATTGCATACGTTGTATCC |
|  |  | 1055 | CGCATGCGCGCACCCTCGATCTCGTATCATCATACCTCAGTTCTTTCCCACCCTTAAGCC |
| pKSB2-H35-∆E1-CG- E4orf6 | galK cassette | 1048 | TGTTCTGCCAGGGCCGCGTTTTGCTATACAAAATACGAGACTGTTGACAATTAATCATCGGCA |
|  |  | 1049 | TCCAACTGCTGCGGATGCGACTCCGGAGTTTGGATCACGGCTCAGCACTGTCCTGCTCCTT |
|  | H5  E4orf6 | 1050 | TGTTCTGCCAGGGCCGCGTTTTGCTATACAAAATACGAGAATGACTACGTCCGGCGTTCC |
|  |  | 1051 | TCCAACTGCTGCGGATGCGACTCCGGAGTTTGGATCACGGCTACATGGGGGTAGAGTCAT |
|  | Non-targeting |  | ACGGAGGCTAAGCGTCGCAA |
|  | Itgb8 #2 |  | GGCATAGTGGTGCCGAATGA |
