# Supplementary material for: The RGD-binding integrins αvβ6 and αvβ8 are receptors for mouse adenovirus-1 and -3 infection
Source: PLoS Pathog. 2021 Dec 15;17(12):e1010083. doi: 10.1371/journal.ppat.1010083 (PMC8673666; doi:10.1371/journal.ppat.1010083)
Supplement: S3 Table — (DOCX) [file ppat.1010083.s023.docx]

S3 Table. Recombinant protein data and oligonucleotides used for generation of expression constructs

| pET28a construct | cloning site | protein sequence | Mr  unmodified  protein  (kDa) | Mr  his-protein  (kDa) | Oligo  Nr | Oligo-Sequence (5’-3’) |
| --- | --- | --- | --- | --- | --- | --- |
| E1A-M1 | EcoRI and XhoI | 1-253 | 28.1 | 31.9 | 2101 | ATATGAATTCATGTCGCGGCTCCTACGTCTTTC |
|  |  |  |  |  | 2102 | GTACCTCGAGCTACCCTAACCACTCCGCCG |
| E1B-19K-M1 | EcoRI and NotI | 1-175 | 20.0 | 23.9 | 2184 | ATATGAATTCATGTTACCTGTGTATCCTTTC |
|  |  |  |  |  | 2185 | ATGCATGCGCGGCCGCTCAAGCTGAGCTCTCTTGCTC |
| IX-M1 | EcoRI and NotI | 1-96 | 11.1 | 14.95 | 2186 | ATATGAATTCATGAATAGCCGTATGCGACGG |
|  |  |  |  |  | 2187 | ATGCATGCGCGGCCGCTCAATCACTTTCTTCCCCGTT |
| FK-M1 | EcoRI and NotI | 361 -613 | 28.1 | 31.9 | 2028 | aattGAATTCaaccaacatggacaattggg |
|  |  |  |  |  | 2029 | gcatgcatGCGGCCGCttaatagtcttcagcatag |
| E1A-M2 | EcoRI and XhoI | 1-263 | 29.0 | 32.8 | 1795 | GTACCTCGAGTCAGGAGTGGCGCTCCACGAAC |
|  |  |  |  |  | 2038 | ATATGAATTCATGGGTGAGAGAGTGTTAAAC |
| FK-M2v1 | EcoRI and NotI | 557 -787 | 25.1 | 28.9 | 2026 | AATTGAATTCCTCAGCGTCACCGTGGAAG |
|  |  |  |  |  | 2027 | CGATCGATGCGGCCGCTTACACGATTCCGATCTGTGAC |
| FK-M2v3 | EcoRI and NotI | 517-787 | 29.5 | 33.3 | 2933 | AATTGAATTCGGCCTCTCCTTCAACGCCG |
|  |  |  |  |  | 2027 | CGATCGATGCGGCCGCTTACACGATTCCGATCTGTGAC |
| E1A-M3 | EcoRI and XhoI | 1-187 | 20.5 | 24.3 | 2103 | ATATGAATTCATGTCTCGTTTACTCCGATTATC |
|  |  |  |  |  | 2104 | GTACCTCGAGTTACTCTTCCTGGAAGGCCC |
| FK-M3 | EcoRI and NotI | 327-567 | 27.0 | 30.8 | 2031 | aattGAATTCaacaaacaaggtcaactagg |
|  |  |  |  |  | 2033 | atgcatgcGCGGCCGCttaatagtcttctgccagatac |
|  |  |  |  |  |  |  |
| pET20b construct | cloning site | protein sequence | Mr  natural  protein  (kDa) | Mr  his-protein  (kDa) | Oligo  Name | Oligo-Sequence (5’-3’) |
| FKb-M1 | BamHI and XhoI | 361 -613 | 28.1 | 31.3 | 3043 | cgcGGATCCaaccaacatggacaattggg |
|  |  |  |  |  | 3044 | ccggCTCGAGttaatagtcttcagcatagtaccaaaaac |
| FKb-M3 | BamHI and XhoI | 327-567 | 27.0 | 30.2 | 3046 | cgcGGATCCaacaaacaaggtcaactaggcctttc |
|  |  |  |  |  | 3047 | ccggCTCGAGttaatagtcttctgccagataccagaaactg |
