# Supplementary material for: The RGD-binding integrins αvβ6 and αvβ8 are receptors for mouse adenovirus-1 and -3 infection
Source: PLoS Pathog. 2021 Dec 15;17(12):e1010083. doi: 10.1371/journal.ppat.1010083 (PMC8673666; doi:10.1371/journal.ppat.1010083)
Supplement: S4 Table — (DOCX) [file ppat.1010083.s024.docx]

S4 Table. Target-template sequence identity.

|  | mouse αv | mouse β6 | mouse β8 | FK-M1 | FK-M3 | FMDV2 |
| --- | --- | --- | --- | --- | --- | --- |
| 5ffo | 0.924 | 0.962 | 0.487 | 0.300 | 0.300 | 0.200 |
| 6uja | 0.934 | 0.311 | 0.654 | 0.300 | 0.300 | 0.200 |
| 4wk0 | 0.405 | 0.290 | 0.263 | - | - | - |
