# Supplementary material for: The RGD-binding integrins αvβ6 and αvβ8 are receptors for mouse adenovirus-1 and -3 infection
Source: PLoS Pathog. 2021 Dec 15;17(12):e1010083. doi: 10.1371/journal.ppat.1010083 (PMC8673666; doi:10.1371/journal.ppat.1010083)
Supplement: S1 Text — (DOCX) [file ppat.1010083.s025.docx]

**Cell library screening**

Two screens were performed using different methods. In a first screen, cytofluorometric analysis of cells infected with the M1-∆E1A-G reporter virus was performed, including 37 rodent (mainly mouse) and 113 human cell lines. For this purpose, triplicates of 10^5^ cells were seeded in 12-well plates. After incubation for 8 h at 37°C, 5% CO_2_, the cells were continuously infected with M1-∆E1A-G reporter virus at three virus concentrations corresponding to MOI 1, 4 and 16. Cells were harvested and processed for cytofluorometric analysis on day 2, 3 and 4, respectively. The ratios of mean GFP expression levels of infected cells and uninfected cells was determined and are available on request.

For the second screen using the M1-∆E1A-G and M3-∆E1A-G reporter viruses, a high throughput imaging microscopy-based method was applied, including 87 mouse cell lines. These were in part overlapping with the first screen and composed of 44 “standard” mouse cell lines including amongst others 8 fibroblast, 4 MEFs, 3 colon, 4 melanoma, 11 hematopoetic, and 2 glioma cell lines. Another 43 cell lines consisted of a collection of more recently immortalized cell lines derived from various different tissues of *Mus musculus*, *Apodemus agrarius*, *Myodes glareolus* and *Sigmodon hispidus* obtained from Isabella Eckerle/Christian Dorsten, Institute of Virology, University of Bonn, Sigmund-Freud-Strasse 25, 53127 Bonn, Germany. The cells were derived from lung (trachea), colon, spleen and kidney epithelia.

The screening conditions were determined using 3T6 cells. Subsequently, for the screen, cell seeding numbers were determined for each of the screened cell lines to get an 80% confluent monolayer at 1 day post seeding. Between 5 x 10^3^ and 6 x 10^4^ cells/well were seeded in in 50 μl medium in 96-well μClear back flat-bottom polystyrene plates (Greiner bio one (655090)). Infection was started 3 h later by adding in an equal volume of 50 μl virus solution. Imaging was performed 5 and 7 days p.i. using an ImageXpress Micro XLS High-Content Imaging System. Data were acquired using the 4x objective with 4 sites per well to obtain the entire well with GFP (300 ms) as well as a transmission light (TL20 20 ms) acquisition. Data was analyzed using the Plaque 2.0 software as well as ImageJ based analyses. Graphs were made using GraphPad Prism 6 for Mac OS X. Total integrated GFP intensities of biological duplicate measurements were normalized against the negative control for each cell line and their distribution was plotted. The screening data are deposited at Mendeley Data.
