# Supplementary material for: The RGD-binding integrins αvβ6 and αvβ8 are receptors for mouse adenovirus-1 and -3 infection
Source: PLoS Pathog. 2021 Dec 15;17(12):e1010083. doi: 10.1371/journal.ppat.1010083 (PMC8673666; doi:10.1371/journal.ppat.1010083)
Supplement: S2 Text — (DOCX) [file ppat.1010083.s026.docx]

**S2 Text. Generation recombinant viruses.**

All nine novel recombinant viruses were generated similarly as described previously using homologous recombination according to the Warmings recombineering protocols 1 and 3 [1,2]. First, this required the generation of the starting bacterial artificial chromosome vectors (BACmid) containing the individual full-length viral genomes, followed by two or more additional steps of recombineering to introduce the sequence modifications of interest. Primers and cloning sites for the individual constructs are listed in S2 Table. Rescue of human recombinant viruses was accomplished by transfecting human 911 cells with unique flanking sites-restricted viral BACmid DNAs using jetPEI (Polyplus Transfection reagents) as described earlier [1]. Initial attempts to rescue infectious MAdV using restricted DNA was not very successful, therefor all recombinant MAdVs, except M1-∆E1A-G, were rescued from additionally generated pKSB2-SceI BACmid constructs, in combination with I-SceI expressing 3T6, CMT-93 or NMU-MG-Fucci cells (see below and [3]). The circular viral BACmid DNAs used in this system were transfected using jetPEI in combination with 1.5 µM 4-OH-tamoxifen treatment 3-5 hours prior to transfection to induce nuclear translocation of ER-I-SceI-HA.

***M1-∆E1A-G, M1-IX-G***

Generation of the bacterial artificial chromosome vector pKSB2-M1 was started with pKSB2-M1-LARAzeo, containing left and right terminal M1 fragments. For this, two PCR-generated fragments were first cloned into pBluescript (pBl). The first fragment encompassed 355 bp of the M1 left end sequence and was PCR-amplified using the primer pair 939/940. The second fragment encompassed 537 bp of the M1 right end sequence and was PCR-amplified using the primer pair 941/942. This second fragment was cloned making use of the XbaI and BamHI restriction sites. A SpeI-fragment containing the zeocin resistance marker from pcDNA3.1 zeo (Invitrogen) and generated by PCR using the primer pair 874/875 was cloned into the SpeI of pBl-M1-LARA, connecting the two M1 arms and resulting in pBl-M1-LARAzeo. Subsequently, the NotI-BamHI-restricted M1-LARAzeo cassette was ligated with the accordingly restricted pKSB2 vector to generate pKSB2-M1-LARAzeo. Colonies containing pKSB2-M1-LARAzeo were selected using chloramphenicol and zeocin at concentrations of 10 µg / ml and 25 µg / ml, respectively. In order to generate pKSB2-M1, homologous recombination was performed in *E. coli* SW102 bacteria, using pKSB2-M1-LARAzeo digested with ApaLI and AatII, which both cut within the zeo cassette, and M1 genomic DNA.

To generate pKSB2-M1-∆E1A-G, the sequence encompassing nucleotide 281 to 1010 was removed including E1A exon 1 and 2 according to sequence annotation by Ball et al. [4], and replaced first with a *galK* cassette, followed by replacement with the GFP ORF sequence. The *galK* cassette and GFP amplicons were PCR-amplified using the primer pairs 947/952 and 950/951, respectively. The introduced sequence consisted of the GFP ORF plus the 3’-untranslated region containing a poly A signal derived from pAdApteGFP which was used as PCR template [5,6]. Rescue of infectious virus from this construct was accomplished following mouse 3T6 cell transfection with PmeI-digested pKSB2-M1-ΔE1A-G DNA.

For the generation of pKSB2-SceI-M1-IX-G, an intermediate pKSB2-M1-IX-G BACmid was first made. For this purpose, the *galK* cassette was inserted into pKSB2-M1, followed by replacement with the 2A-GFP cassette downstream of protein IX gene using *galK* and 2A-GFP amplicons produced with primer pair 2325/2326 and 2327/2553, respectively. For the second PCR, pBl-2A-GFP was used as template, which itself was generated by PCR using the primer pair 2305/2306 introducing the 4 amino acid residues of the furin cleavage site and the 24 amino acid residues of the 2A auto processing site derived from the food and mouth disease virus [7] fused to the N-terminal end of GFP.

In order to generate the SceI-modified pKSB2-SceI-M1-IX-G, a modified version of pKSB2 was first generated containing two copies of the 18 nucleotide I-SceI recognition sequence [3] flanking the multiple cloning site of pKSB2. For this purpose, a PCR product containing a *KanR* cassette was generated, using the primer pair 2371/2372 introducing a BstEII site and the pair of I-SceI sites into the pKSB2 multiple cloning site. The BstEII-digested PCR DNA was cloned into the BstEII and HindIII (blunt) of pKSB2 resulting in pKSB2-SceI-KanR. The NotI-BamHI fragment containing the M1-LARAzeo fragment (see above) was subsequently cloned into pKSB2-SceI-KanR to generate pKSB2-SceI-M1-LARAzeo. Finally, in a further recombineering step, the M1-IX-G fragment obtained by PmeI digest of pKSB2-M1-IX-G was recombined to the pKSB2-SceI-M1-LARAzeo fragment obtained after ApaLI and AatII double digest. Using circular BACmid DNA of the resulting pKSB2-SceI-M1-IX-G, infectious virus was rescued from transfected mouse CMT-93 as well as 3T6 cells stably expressing I-SceI endonuclease.

***M2-∆E1A-G***

Generation of pKSB2-SceI-M2-∆E1A-G was performed stepwise as described above. pBl-M2-LARAzeo containing 502 bp of the M2 left end sequence, 465 bp of the M2 right end sequence, and the zeo cassette were ligated from fragments generated using primer pair 1320/1321, 1322/1323 and 874/875, respectively. pKSB2-M2-LARAzeo was generated by transfer of the NotI-BamHI-restricted M2-LARAzeo cassette to the accordingly restricted pKSB2 vector. To generate pKSB2-M2, homologous recombination was performed in *E. coli* SW102 bacteria, using pKSB2-M2-LARAzeo digested with ApaLI and AatII and M2 genomic DNA.

To generate pKSB2-M2-∆E1A-G the sequence encompassing nucleotide 348 to 975 was removed corresponding to E1A exon 1 [8], and replaced first with a *galK* cassette, followed by replacement with the GFP ORF sequence. The *galK* cassette and GFP amplicons were PCR amplified using the primer pairs 1597/1598 and 1717/1718, respectively. Since no virus was rescued from the construct following PacI digest in 3T6 cells, pKSB2-SceI-M2-∆E1A-G was generated. For this, the NotI-BamHI fragment containing the M2-LARAzeo fragment was cloned into pKSB2-SceI-KanR to generate pKSB2-SceI-M2-LARAzeo. Finally, in a further recombineering step, the M2-∆E1A-G fragment obtained by PacI digest of pKSB2-M2-∆E1A-G was recombined to the pKSB2-SceI-M2-LARAzeo fragment obtained after ApaLI and AatII double digest. Using circular BACmid DNA of the resulting pKSB2-SceI-M2-E1A-G, infectious virus was rescued in mouse NMU-MG-I-SceI cells.

***M3-∆E1A-G, M3-IX-G***

Generation of the bacterial artificial chromosome vector pKSB2-SceI-M3-∆E1A-G was started with first cloning pBl-M3-LARAzeo containing left and right terminal M3 800 bp fragments. For this purpose, the two PCR were performed using the primer pairs 2090/2091 and 2092/2093, respectively. The zeo cassette separating the left arm–right arm sequences was inserted as described for M1 and M2. Transfer of the M3-LARAzeo cassette to generate pKSB2-M3-LARAzeo was accomplished using the cloning sites NsiI-HindIII. This was followed by homologous recombination to generate pKSB2-M3. To generate the intermediate BACmid pKSB2-M3-∆E1A-G, the *galK* cassette produced by PCR using the primer pair 2125/2126 was placed in the E1A exon 1 sequence from nucleotide 255 to 720. Subsequently, the *galK* was replaced with a 2127/2128 primer pair PCR product as described for M1.

In order to generate pKSB2-SceI-M3-∆E1A-G, the intermediate construct pKSB2-SceI-M3-LARAzeo was cloned by ligating pKSB2-SceI-KanR and the above described pBl-M3-LARAzeo using the NsiI and HindIII sites. Subsequently, using recombineering, the M3-∆E1A-G fragment obtained by PacI digest of pKSB2-M3-∆E1A-G was fused to the pKSB2-SceI-M3-LARAzeo fragment obtained after ApaLI and BamHI digest. Infectious virus was rescued in mouse NMU-MG-I-SceI cells.

For the generation of pKSB2-SceI-M3-IX-G, we first generated pKSB2-SceI-M3 similarly as described for M1 and-2. Insertion of the *galK* cassette, followed by replacement with the 2A-GFP cassette downstream of protein IX gene was accomplished using *galK* and 2A-GFP amplicons produced with primer pairs 2556/2557 and 2560/2561, respectively. Infectious virus was rescued in mouse NMU-MG-I-SceI cells.

***H5-∆E3B-CG, H5-∆E3B-CG-FK-M1, H5-∆E3B-CG-FK-M3***

As a first step, a pKSB2-based bacterial artificial chromosome vector carrying the H5 genome (wt300) [9] was generated starting with pKSB2-H5-LARAzeo containing left and right terminal H5 fragments. To generate pKSB2-H5-LARAzeo, two PCR-generated fragments were first cloned into pBl. The first fragment encompassed 425 bp of the H5 left end sequence and was PCR-amplified using the primer pair 943/958 and cloned into into pBl. The second fragment encompassed 408 bp of the H5 right end sequence and was PCR-amplified using the primer pair 945/946 and inserted into pBl-H5-LA. The SpeI fragment containing the zeocin resistance marker was inserted as described for M1, connecting the two H5 arms and resulting in pBl-H5-LARAzeo. In order to transfer the H5-LARAzeo cassette to the BACmid pKSB2, the NotI-HindIII fragment containing this sequence was ligated with the NotI-HindIII-restricted pKSB2 vector. Colonies containing pKSB2-H5-LARAzeo were selected using chloramphenicol and zeocin at concentrations of 10 µg/ml and 25 µg/ml, respectively. In order to generate pKSB2-H5, homologous recombination was performed in SW102 bacteria using AatII and ApaLI-restricted pKSB2-H5-LARAzeo and H5 genomic DNA.

For the generation of pKSB2-H5-∆E3B-CG, insertion of the *galK* cassette, followed by replacement with the CMV-GFP-pA cassette was accomplished using *galK* and CMV-GFP cassette amplicons produced with primer pairs 2910/2911 and 2912/2913, respectively. This led to deletion of 1065 nucleotides, removing the E3B genes, similarly as described in [10].

For the generation of pKSB2-H5-∆E3B-CG-FK-M1, insertion of the *galK* cassette was followed by replacement with the FK-M1 sequence encompassing nucleotide residues 1159-1842 of M1 fiber. This was accomplished using *galK* and FK amplicons produced with primer pairs 2903/2900 and 2904/2902, respectively. For pKSB2-H5-∆E3B-FK-M3, *galK* was replaced with the PCR product from primer pair 2917/2918 with the FK-M3 sequence encompassing nucleotide residues 1057-1704 of M3 fiber. Both fiber-chimeric viruses were rescued following transfection of PacI-digested DNA in 911 cells expressing inducible lung macrophage scavenger receptor SR-A6 ([11] and unpublished data).

***H35-∆E1-CG***

As a first step, a pKSB2-based bacterial artificial chromosome vector carrying the H35 genome was generated starting with pKSB2-H35-LARAzeo containing left and right terminal H35 fragments. To generate pKSB2-H35-LARAzeo, two PCR-generated fragments were first cloned into pBl. The first fragment encompassed 617 bp of the H35 left end sequence and was PCR-amplified using the primer pair 1040/1041 and cloned into into pBl. The second fragment encompassed 625 bp of the H35 right end sequence and was PCR-amplified using the primer pair 1042/1043 and inserted into pBl-H35-LA. The SpeI fragment containing the zeocine resistance marker was inserted as described for M1, connecting the two H35 arms and resulting in pBl-H35-LARAzeo. In order to transfer the H35-LARAzeo cassette to the BACmid pKSB2, the NotI-HindIII fragment containing this sequence was ligated with the NotI-HindIII-restricted pKSB2 vector. Colonies containing pKSB2-H35-LARAzeo were selected using chloramphenicol and zeocin at concentrations of 10 µg/ml and 25 µg/ml, respectively. In order to generate pKSB2-H35, homologous recombination was performed in SW102 bacteria using AatII and ApaLI-restricted pKSB2-H35-LARAzeo and H35 genomic DNA.

As a second step, pKSB2-H35-∆E1-CG-E4orf6 was generated using two recombineering steps. As a first, the *galK* cassette was introduced into pKSB2-H35 to replace the E1 sequence from nt 465 to 3232. The *galK* cassette was amplified using the primer pair 1052/1053. As a second, the CMV-GFP cassette was PCR-amplified using the primer pair 1054/1055 and pAdApt.eGFP as template. The resulting deletion comprised the ORFs for E1A and most of E1B, but left intact a residual 251-bp stretch of the protein IX promoter [12].

As a third step, the replacement of the endogenous H35 E4-orf6 in pKSB2-H35-∆E1-CG with the E4-orf6 of H5 was accomplished in a further recombineering step. The *galK* cassette was introduced into pKSB2-H35-∆E1-CG-E4orf6 to replace the H35 E4-orf6 sequence using a PCR *galK* amplicon generated with the primer pair 1048/1049. Subsequently the *galK* cassette was replaced by the H5 E4-orf6 sequence amplified using the primer pair 1050/1051. Release of the viral genome by the flanking unique Pme I endonuclease sites was followed by transfection of 911 cells.

**References**

1. Sirena D, Ruzsics Z, Schaffner W, Greber UF, Hemmi S. The nucleotide sequence and a first generation gene transfer vector of species B human adenovirus serotype 3. Virology. 2005;343(2):283-98. PubMed PMID: 16169033.

2. Warming S, Costantino N, Court DL, Jenkins NA, Copeland NG. Simple and highly efficient BAC recombineering using galK selection. Nucleic Acids Res. 2005;33(4):e36. PubMed PMID: 15731329.

3. Ibanes S, Kremer EJ. Canine adenovirus type 2 vector generation via I-Sce1-mediated intracellular genome release. PLoS One. 2013;8(8):e71032. doi: 10.1371/journal.pone.0071032. PubMed PMID: 23936483; PubMed Central PMCID: PMCPMC3731271.

4. Ball AO, Beard CW, Redick SD, Spindler KR. Genome organization of mouse adenovirus type 1 early region 1: a novel transcription map. Virology. 1989;170(2):523-36. Epub 1989/06/01. PubMed PMID: 2543128.

5. Holterman L, Vogels R, van der Vlugt R, Sieuwerts M, Grimbergen J, Kaspers J, et al. Novel replication-incompetent vector derived from adenovirus type 11 (Ad11) for vaccination and gene therapy: low seroprevalence and non-cross-reactivity with Ad5. J Virol. 2004;78(23):13207-15. PubMed PMID: 15542673.

6. Fleischli C, Sirena D, Lesage G, Havenga MJ, Cattaneo R, Greber UF, et al. Species B adenovirus serotypes 3, 7, 11 and 35 share similar binding sites on the membrane cofactor protein CD46 receptor. J Gen Virol. 2007;88(Pt 11):2925-34. PubMed PMID: 17947513.

7. Robinson M, Li B, Ge Y, Ko D, Yendluri S, Harding T, et al. Novel immunocompetent murine tumor model for evaluation of conditionally replication-competent (oncolytic) murine adenoviral vectors. J Virol. 2009;83(8):3450-62. Epub 2009/02/06. doi: JVI.02561-08 [pii]

10.1128/JVI.02561-08. PubMed PMID: 19193803; PubMed Central PMCID: PMC2663273.

8. Hemmi S, Vidovszky MZ, Ruminska J, Ramelli S, Decurtins W, Greber UF, et al. Genomic and phylogenetic analyses of murine adenovirus 2. Virus Res. 2011;160(1-2):128-35. doi: 10.1016/j.virusres.2011.05.023. PubMed PMID: 21683742.

9. Hearing P, Shenk T. The adenovirus type 5 E1A transcriptional control region contains a duplicated enhancer element. Cell. 1983;33(3):695-703. PubMed PMID: 6871991.

10. Yakimovich A, Gumpert H, Burckhardt CJ, Lutschg VA, Jurgeit A, Sbalzarini IF, et al. Cell-free transmission of human adenovirus by passive mass transfer in cell culture simulated in a computer model. J Virol. 2012;86(18):10123-37. doi: 10.1128/JVI.01102-12. PubMed PMID: 22787215; PubMed Central PMCID: PMCPMC3446567.

11. Stichling N, Suomalainen M, Flatt JW, Schmid M, Pacesa M, Hemmi S, et al. Lung macrophage scavenger receptor SR-A6 (MARCO) is an adenovirus type-specific virus entry receptor. PLoS Pathog. 2018;14(3):e1006914. doi: 10.1371/journal.ppat.1006914. PubMed PMID: 29522575; PubMed Central PMCID: PMCPMC5862501.

12. Havenga M, Vogels R, Zuijdgeest D, Radosevic K, Mueller S, Sieuwerts M, et al. Novel replication-incompetent adenoviral B-group vectors: high vector stability and yield in PER.C6 cells. J Gen Virol. 2006;87(Pt 8):2135-43. PubMed PMID: 16847108.
