# Supplementary material for: The RGD-binding integrins αvβ6 and αvβ8 are receptors for mouse adenovirus-1 and -3 infection
Source: PLoS Pathog. 2021 Dec 15;17(12):e1010083. doi: 10.1371/journal.ppat.1010083 (PMC8673666; doi:10.1371/journal.ppat.1010083)
Supplement: Attachment — Submitted filename: Bieri_Rebuttal.docx [file ppat.1010083.s027.docx]

Dear Editor

we would like to thank the editor and the reviewers for their positive assessment of our manuscript. Below, please find our point-by-point response to the reviewers’ comments.

Reviewer's Responses to Questions

**Part I - Summary**
Please use this section to discuss strengths/weaknesses of study, novelty/significance, general execution and scholarship.

Reviewer #1: Hemmi et al. have identified and characterized the receptors mediating infection of the mouse adenovirus (MAdV) using novel recombinant mouse viruses. They determine that RGD-recognizing ⍺vß6 and ⍺vß8 integrin heterodimers are the receptors for MAdV types M1 and M3. This is based on six different experimental criteria including studies in permissive/nonpermissive cell lines, gain-of-function in non-permissive cells, antibody-mediated blocking, synthetic peptide-mediated blocking, CRISPR/shRNA knockout/knockdown studies and SPR analysis. They provide substantial associative, deductive and experimental evidence to support their findings. The studies are rigorously performed, thoroughly reported and are of suitable novelty to merit publication in PLOS Pathogens (provided they address the minor comments below).

Reviewer #2: This study identifies a cell entry receptor for mouse adenoviruses MAdV M1 and M3. Little is known about entry of murine adenoviruses, and identification of receptors would expand our understanding of infection and could have implications for gene therapy vector delivery. A previous report from the Spindler group suggested that alpha-v integrins and heparan sulfate are used. The authors here focus on integrins as potential receptors for M1 and M3 and they start by generating new reporter viruses. They used these viruses to screen cells for infection. Recombinant fiber knob and antisera blocked transduction by these reporter viruses and suggested similar receptor usage for M1 and M3. They show that the CAR receptor is not used and then demonstrate that overexpression of integrins enhanced binding and infection. Anti-integrin antibodies blocked M1 and M3 binding and infection. Infection was also blocked by soluble integrin, and RGDLXXL peptides. They also showed that genetic depletion of integrins reduced infection. Direct binding of fiber to integrins was demonstrated by surface plasmon resonance and cell binding. Finally, molecular docking simulations support that the RGD sequence in the fiber protein interacts with the mouse integrin chain. They conclude that the RGDL motif in the fiber knob is important for M1 and M3 infection through the conserved alpha-v-beta-6/8 integrins. The work is comprehensive, rigorous and compelling. The Introduction and Discussion are well written, but the Results section is a little challenging to work through. Overall, this is a very nice contribution to the field, and I have little to suggest for improvement.

**Part II – Major Issues: Key Experiments Required for Acceptance**
Please use this section to detail the key new experiments or modifications of existing experiments that should be absolutely required to validate study conclusions.

Generally, there should be no more than 3 such required experiments or major modifications for a "Major Revision" recommendation. If more than 3 experiments are necessary to validate the study conclusions, then you are encouraged to recommend "Reject".

Reviewer #1: I have no major comments or concerns about this manuscript.

Reviewer #2: Having identified the RGD motif in the fiber as important for interaction with integrins, the work could be further strengthened by showing that mutant peptides cannot bind to the integrin in the SPR and cell binding studies of Figure 7, and also cannot inhibit transduction. This would close the loop and further support their conclusions.

Author response: The reviewer requests a series of additional experiments. To fulfill this request, we would need to generate new bacterial expression constructs and prepare new recombinant biotinylated fiber knob proteins FK-M1 and FK-M3, each containing RGD to RGE mutations. Experiments to be repeated with at least two new proteins would be at least three-fold, and involve (1) SPR measurements of FK binding to one or maximal four integrins, (2) FK cell binding studies with various cell lines, and (3) virus transduction blocking studies. We would anticipate a major delay of 4-6 months until we would be ready to submit a revised manuscript.

It is questionable in our opinion, if these efforts were justified, in the light of the fact that it is a control experiment with an expected negative effect on binding and infection. As the reviewer points out, it would at best serve to ‘close the loop’: ‘Having identified the RGD motif in the fiber as important for interaction with integrins, the work could be further strengthened by showing that mutant peptides cannot bind to the integrin in the SPR and cell binding studies of Figure 7, and also cannot inhibit transduction. This would close the loop and further support their conclusions.’

In fact, the reviewer’s comment implies that the expected outcome would likely be a negative result. We base this expectation on earlier experiments performed by Ramon et al (ref 43, their Fig 2B). These investigators used recombinant FK-M1 proteins containing an RGD to KGE or AAA mutations, and reported that the mutated FK-M1 no longer blocked the infection of cells with mouse AdV-1. Unfortunately, this protein cannot not be used for the requested experiments, since it lacks the biotinylation feature. Importantly, however, we already provide a similar experiment with mutated RGE peptides, as requested by the reviewer. The data from these experiments are presented in Fig 2G and S9 Fig. They show that the 20mer peptides derived from the FMDV2 virus coat protein with the mutated RGELXXL motif had strongly reduced binding affinity to integrins compared to a peptide bearing the natural RGDLXXL motif.

Considering the wealth of data in our manuscript and our strongly supported conclusions based thereupon (see reviewer #1), the benefit from additional experiments to the central message of the paper is minimal and not justified.

**Part III – Minor Issues: Editorial and Data Presentation Modifications**
Please use this section for editorial suggestions as well as relatively minor modifications of existing data that would enhance clarity.

Reviewer #1: Figure 1A: The actin levels in the immunoblots suggest that the samples are not loaded consistently. At 72 hpi timepoints this might be due to cytopathic effects, but can the authors provide an explanation for the inconsistency in loading at 8 hpi and 14 hpi timepoints for M1-IX-G?
Author response: The reviewer is correct, there was a mistake in this actin blot of Fig1A. It consisted of an additive staining of anti-GFP and anti-actin and showed in addition to actin also the unprocessed IX-2A-GFP. We apologize for this. The blot has been exchanged with an appropriate blot of the same samples.

Figure 1B, 1C: Although the authors note that the data presented is in triplicate and are presented as mean +/- SEM, there are no error bars or statistics visible on these graphs.
Author response: This is due to the combination of small error bars in this type of experiments and large marker style symbols; in fact, a data point with large error bar is visible for M3-IX-G infection (Fig 1C, % positive cells) at the forth time point.

Line 115-117: This seems like a contradictory statement. Please restate or clarify
Author response: we think the statement is correct. Be aware, in the first sentence we allude to penton base, and in the second sentence to fiber.

Figure 2: While the trendlines and error bars are somewhat compelling, the authors should provide information on statistical significance of this datasets to remove any ambiguity around the conclusions.
Author response: thank you for this comment. We now indicated p values for the highest concentrations of FKs and anti-FK sera. A sentence was added to the Fig legend: ‘For highest concentrations of FKs and anti-FK sera, asterisks indicate level of significance for comparison of indicated values (*, *P*<0.05; **, *P*<0.005; ***, *P*<0.0005); ns: not significant (*P*>0.05).’

We also added a sentence in the Results section to comment the unexpected weak trend found for FK-M1/-M3 to block at highest concentration infection of M2-∆E1A-G: ‘The highest concentrations of the FK-M1/-M3 weakly inhibited M2-∆E1A-G infection, which currently remains unexplained.’

Figure 5B/C: Please consider spacing out the labels containing the ion names (Mg/CaMn/Ca..etc) to increase clarity.
Author response: thank you. The figure has been modified in 5B/C/D and 6A.

Where is figure 6J? The figure and labels jump from 6I to 6K.
Author response: thank you, this error has been corrected in Fig 6, as well as in S14 Fig.

Line 504: “undermined”, as stated in the manuscript means “to erode”. I think the authors mean “underlined” (meaning, “to highlight”).
Author response: this has been corrected

Line 607: misspelled “criteria”
Author response: this has been corrected

Reviewer #2: 1) The recombinant viruses generated are useful tools for the field. It would be helpful to include maps for the recombinant viruses generated in Table 1.
Author response: as per request of the reviewer, we have now added these schematics as S1 and S2 Figs representing maps of novel mouse and human recombinant viruses, respectively. Two new legends were added and S Fig numbering was adjusted.

2) The Introduction is well written, but I suggest one addition. The definition of a viral receptor is sometimes controversial. I suggest the authors add a section in which they provide their definition for a viral receptor and the criteria that need to be met. They then can highlight how they go onto to fulfill all these requirements.
Author response: we have added a new section at the end of the introduction:

Here, we adopted a stringent definition of a virus receptor, contrasting attachment factors and facilitators {Yamauchi, 2016, 26875443}. We consider a receptor to be a protein, sugar or lipid molecule, which makes direct physical contact to a virion component on the cell surface, and thereupon triggers the uptake of the virus particle (or at least the viral genome) into the cell, followed by either infection or inactivation. Receptors stand in contrast to attachment factors, which can bind to the virus particle but do not lead to infection or virion clearance. They also stand in contrast to a third class of cell surface molecules, so called facilitators, which assist the function of receptors for the virion. These distinctions are increasingly important, as the cell-to-cell variability of virus infections involves not just the cell internal state {Suomalainen, 2020 #3274}, but also the abundance of cell surface molecules and their interactions with virions {for a recent review, see \Suomalainen, 2021, 34452433}. To fulfill stringent receptor criteria, we used viral and host genetic engineering, biochemical and cell biological assays as well as molecular modeling, and demonstrate that M1/M3 use RGD-motifs in their FKs to bind with high affinity to αvβ6 and αvβ8 integrins for infection of murine and human cells.

We appreciate your time for looking into this, and hope the manuscript is now acceptable to PloS Pathogens.

Yours, sincerely,

Silvio Hemmi, PhD

University of Zürich

Department of Molecular Life Sciences

PLOS authors have the option to publish the peer review history of their article ([what does this mean?](https://journals.plos.org/plospathogens/s/editorial-and-peer-review-process#loc-peer-review-history)). If published, this will include your full peer review and any attached files.


If you choose “no”, your identity will remain anonymous but your review may still be made public.


**Do you want your identity to be public for this peer review?** For information about this choice, including consent withdrawal, please see our [Privacy Policy](https://www.plos.org/privacy-policy).

Reviewer #1: No

Reviewer #2: **Yes:** Matthew D. Weitzman

Figure Files:

While revising your submission, please upload your figure files to the Preflight Analysis and Conversion Engine (PACE) digital diagnostic tool, https://pacev2.apexcovantage.com. PACE helps ensure that figures meet PLOS requirements. To use PACE, you must first register as a user. Then, login and navigate to the UPLOAD tab, where you will find detailed instructions on how to use the tool. If you encounter any issues or have any questions when using PACE, please email us at figures@plos.org.

Data Requirements:

Please note that, as a condition of publication, PLOS' data policy requires that you make available all data used to draw the conclusions outlined in your manuscript. Data must be deposited in an appropriate repository, included within the body of the manuscript, or uploaded as supporting information. This includes all numerical values that were used to generate graphs, histograms etc.. For an example see here: <http://www.plosbiology.org/article/info%3Adoi%2F10.1371%2Fjournal.pbio.1001908#s5>.

Reproducibility:

To enhance the reproducibility of your results, we recommend that you deposit your laboratory protocols in protocols.io, where a protocol can be assigned its own identifier (DOI) such that it can be cited independently in the future. Additionally, PLOS ONE offers an option to publish peer-reviewed clinical study protocols. Read more information on sharing protocols at <https://plos.org/protocols?utm_medium=editorial-email&utm_source=authorletters&utm_campaign=protocols>

References:

Please review your reference list to ensure that it is complete and correct. If you have cited papers that have been retracted, please include the rationale for doing so in the manuscript text, or remove these references and replace them with relevant current references. Any changes to the reference list should be mentioned in the rebuttal letter that accompanies your revised manuscript. If you need to cite a retracted article, indicate the article’s retracted status in the References list and also include a citation and full reference for the retraction notice.
